# Pressure-Stable Imprinted Polymers for Waste Water Remediation

**DOI:** 10.3390/polym10070704

**Published:** 2018-06-26

**Authors:** Shane Mann, Travis Johnson, Evie Medendorp, Robert Ocomen, Luke DeHart, Adam Bauer, Bingbing Li, Mary Tecklenburg, Anja Mueller

**Affiliations:** 1 Department of Chemistry and Biochemistry, Central Michigan University, Mount Pleasant, MI 48859, USA; mann2sc@cmich.edu (S.M.); johns24t@cmich.edu (T.J.); eviemedendorp@yahoo.com (E.M.); ocome1rc@cmich.edu (R.O.); dehar1ls@cmich.edu (L.D.); li3b@cmich.edu (B.L.); teckl1mm@cmich.edu (M.T.); 2 Department of Science of Advanced Materials, Central Michigan University, Mount Pleasant, MI 48859, USA; bauer1aj@cmich.edu

**Keywords:** molecularly-imprinted polymer (MIP), heavy metal ions, water remediation, zinc ions, calcium ions, intermolecular bonds, pressure stable, ion imprinted polymers (IIP)

## Abstract

In wastewater treatment, the removal of heavy metal ions is difficult. Ion exchange resins are ineffective since heavy metal ions cannot compete with “hard ions” in binding to the resins. Imprinting polymerization can increase the specificity of ion exchange resins to allow heavy metal ions to compete. Unfortunately, a high capacity is also needed. When high porosity and surface area are used to increase capacity, polymeric resins lose pressure stability needed for water treatment. In this research, a bulky, hydrophobic co-monomer was used to prevent Zn^+2^ imprinted sites from collapsing. Both the co-monomer and crosslinking density were optimized to allow for maximum pore access while maintaining pressure stability. IR and SEM studies were used to study phase separation of the hydrophobic co-monomer from the hydrophilic resin. Capacity was measured for just the imprinting ion first, and then in combination with a competing ion and compared with porosity and pore-size measurements. Capacity under pressure was also characterized. A resin with high capacity was identified that allowed for the heavy metal ion to compete while still maintaining pressure stability.

## 1. Introduction

Molecularly imprinted polymers (MIPs) encompass a wide array of modern applications owing in large part to their inherent versatility. By definition, an MIP is a polymeric, or composite, material that can be synthetically tailored to any chemical application requiring specificity [1,2,3]. In this study, a MIP was designed on the molecular level to remove Zinc ions (Zn^+2^) from water under the high pressure used in water treatment [4,5,6,7].

The basis of the design were ion exchange resins. Industrially, acrylate and acrylamide systems are established within the waste water treatment community, therefore they were used here [4,5,6]. Ion exchange resins based on acrylates bind the more common “hard ions”, Ca^+2^ and Mg^+2^, more strongly than heavy metal ions [1,2,5,6,8]. Copolymerization with acrylamides allows for the formation of metal-ligand bonds, not just the ionic bonds [6,9]. With ion-imprinting, specific sites with the stronger metal-ligand bonds for the heavy metal ions are formed, thus allowing the heavy metal ion to compete for the resin binding sites. The strong metal-ligand forces allow for specificity of binding, but some of the acrylate sites also bind other ions in the water, helping to reduce hardness at the same time. The efficacy of this approach has been demonstrated using resins imprinted with Pb^+2^, Cd^+2^, Hg^+2^, Zn^+2^, Cs^+^, Fe^+3^, Cu^+2^, Ag^+^, Cr^+3^, As^+3^, and Cu^+2^ cations [9,10,11,12,13,14,15,16,17,18,19].

The stability of the imprinted site is usually ensured by using a very high crosslinking density. With that, only binding sites on the surface of the resin particles can be accessed [20]. In that case, capacity can only be increased by decreasing particle size and thus increasing the amount of surface area [9,10,11,12,13,14,15,16,17]. In this study, crosslinking density was greatly reduced, and pore size was additionally enhanced, to allow access to binding sites at the surface as well as throughout the volume of the resin particles [10,11]. This allows for more surface area to be accessed and thus capacity for ion binding to be increased. The exact capacity will still depend on particle size, exact pore size, and tortuosity of the pores. The high porosity, however, reduces the pressure stability of the resin. In this paper, the porous resin is stabilized towards pressure by using a bulky, hydrophobic monomer that keeps the pores from collapsing [9,10,11].

The novel system described herein expands upon the specificity and capacity of previous systems by implementing a *tert*-butyl acrylate (tBA) moiety into the polymeric backbone to achieve pressure stability. *tert*-Butyl acrylate copolymerizes alongside methacylamide and methacrylate. The result is a polymeric structure where *tert*-butyl acrylate groups repel one another, creating rigidity through hydrophobicity, without interfering with, or deforming, the imprinted sites.

In this investigation to create a pressure-stable system, the addition of *tert*-butyl groups to the structure was compared to simply crosslinking the original system more. The crosslinking densities achieved in this study did not achieve pressure stability and strongly reduced capacity, suggesting that access to the pores inside the resin was greatly reduced. The addition of tBA to the structure of the resin did achieve pressure stability. Evidence also suggests that the hydrophobic tBA initiates phase separation at a higher concentration. Phase separation creates hydrophobic pockets that the hydrophilic hydrated ions will not be able to access, thus reducing some of the capacity despite high porosity and surface area. This study reports initial data discussing the balance between binding specificity, porosity and surface area, binding-site access, and capacity, while achieving pressure stability.

All polymers were characterized by infrared spectroscopy, thermogravimetric analysis, and differential scanning calorimetry. Variations in porosity due to changes in crosslinker and tBA concentrations as well as imprinted vs. non-imprinted polymers, were tracked using Brunauer–Emmett–Teller (B.E.T.) surface area and pore size measurements using physisorption equipment.

## 2. Materials and Methods

### 2.1. Materials

The following synthetic materials were obtained from Sigma Aldrich (St. Louis, MO, USA) at the denoted purities: methacrylamide (98%), methacrylic acid (99%), ethylene glycol diacrylate (EGDA, 90%), *tert*-butyl acrylate (98%, 10–20 ppm monomethyl ether hydroquinone), and 2,2-azobis(methyl propionamidine) dihydrochloride (AAPD, 97%). Zinc chloride (ZnCl_2_) was obtained from Fischer Scientific (Pittsburgh, PA, USA) at 99.9% purity. Calcium chloride (CaCl_2_) was obtained from EM Science (Norwood, OH, USA) at 90% purity. Deionized ultrafiltered (DIUF) water was obtained from an E-pure water filtration system (Pittsburgh, PA, USA) and was collected at 18 MOhms.

### 2.2. Synthesis of Non-Imprinted and Zn^+2^ Ion Imprinted Poly(methacrylate co-methacrylamide) for Crosslinking Density Analysis

All polymers were synthesized in a Rayonet RPR-100 500 mL UV photochemical reactor (Branford, CT, USA) by the following procedure, unless stated otherwise: Into the 500 mL UV reactor flask was placed 103.5 mL of DIUF water, 6.31 mL of methacrylic acid (72.6 mmol, 0.7 eq), and 2.65 g of methacrylamide (31.1 mmol, 0.3 eq). A varied amount of crosslinking agent, EGDA (5 mol %, 0.9 mL; 10 mol %, 1.9 mL; 15 mol % 2.7 mL), was added to the mixture followed by 185 mg (6.8 × 10^−6^ eq) of the AAPD initiator. All reagents are water soluble [21,22]. Zinc-imprinted versions of each polymer were also made by adding 135 mg of ZnCl_2_ (1 mol %). This mixture was stirred for 4–6 h while bubbling nitrogen through the reaction to increase resin porosity. The polymer was vacuum filtered. Zinc-imprinted polymers were dialyzed for one week (Section 2.4) until no zinc ions were found in flame AA measurements. Polymers were then dried using a VirTis Advantage ES-53 freeze drier (St. Louis, MO, USA) for 24 h. All polymers were ground using a mortar and pestle and screened through a 500 micron stainless steel wire mesh flour sieve. The yield of the resins can be calculated by monitoring conversion of starting materials in relation to the final weight of the dried resin post lyophilization. However, all polymers gave yields larger than 100% due to their hygroscopic nature. All polymers gave similar spectra via IR analysis: 3500–2750, 3490, 3359, 2988, 1710, 1658, 1476, 1389, 1254, and 1163 cm^−1^ [16,23,24].

### 2.3. Synthesis of Non-Imprinted and Zn^+2^ Ion Imprinted Poly(methacrylate co-methacrylamide) with tBA (Figure 1)

Synthesis of non-imprinted polymers required charging a Rayonet RPR-100 500 mL photochemical reactor (Branford, CT, USA) with 6.31 mL of methacrylic acid (72.6 mmol, 0.7 eq), 2.65 g of methacrylamide (31.1 mmol, 0.3 eq), along with a varied amount of *tert*-butyl acrylate (1 mol %, 0.110 mL; 3 mol %, 0.330 mL; 5 mol % 0.550 mL), 0.962 mL of EGDA (5 mol %, 6.18 mmol), and 103.5 mL of DIUF H_2_O (5.74 mol). The reaction mixture was sparged with nitrogen for 30 min before 0.185 g of the 2,2-azobis(2-methylpropionamidine) dihydrochloride initiator (0.6932 mmol, 0.6 mol %, 6.8 × 10^−6^ eq) was added. The reaction was stirred for 4–6 h while bubbling nitrogen through the reaction to increase resin porosity. The reaction mixture was vacuum filtrated and rinsed with DIUF water to remove residual unreacted starting materials [16].

For imprinted polymers, an additional 135 mg of anhydrous ZnCl_2_ (1 mol %) was added, otherwise all procedures were identical. Dried, imprinted polymers were subjected to dialysis for approximately one week to remove zinc ions (see Section 2.4 for further detail). Post dialysis, all polymers were again vacuum filtered and lyophilized for 72 h using a Labcono FreeZone 1 L benchtop freeze dry system (Kansas City, MO, USA). All polymers were ground using a mortar and pestle and screened through a 500 micron stainless steel wire mesh flour sieve. All polymers gave yields larger than 100% due to their hygroscopic nature; all polymers gave similar spectra via IR analysis: 3500–2750, 3490, 3359, 2988, 1710, 1658, 1476, 1389, 1254, and 1163 cm^−1^ [23,24].

### 2.4. Removal of Zinc Ions via Dialysis

Dialysis was chosen as a non-intrusive method to remove zinc ions without introducing organic solvents to the resins, while simultaneously preserving ion imprinted sites. Zinc-imprinted ion exchange resins underwent one week of dialysis using Fisher brand (Pittsburgh, PA, USA) regenerated cellulose dialysis tubing with a molecular weight cut off (MWCO) of 6000–8000 Daltons. All polymers added as a DIUF slurry to 50–100 cm long sections of cellulose tubing. Individual sections were fully submerged in DIUF water. DIUF water was refreshed once per day until no metal ion absorbance values could be detected via flame AA (see Section 2.12 for further detail). Polymers collected via vacuum filtration and rinsed with DIUIF water, followed by lyophilization.

### 2.5. Pressure Stability Measurements

All pressure experiments were run using an in-house pressure set-up, as shown in Figure 2, using a Fluid Metering Inc. Model QV FMI Lab pump (Syosset, NY, USA) connected to a Fluid Metering V300 Stroke Rate Controller (Syosset, NY, USA) to control the desired pressure. For all polymers, except the 5 mol % EGDA, approximately 1 g of resin was soaked in about 30 mL of DIUF water for 20 min to swell. For the 5 mol % EGDA polymer, 2 g was used. Afterwards, the swollen polymer was rinsed into the pressure column, Figure 2 left image.

A polymer was considered to pass or fail based on whether the pressure relief valve could be fully closed without over-pressurizing the system above 60 psi. Polymers that passed this test were then allowed to compact under pressure for three hours or until flow rate, compression, and pressure readings stabilized. Flow rates were calculated from the collection port. All polymers were placed under a stroke rate of 8% the pump’s maximum, unless otherwise noted. Once the system stabilized, the stroke rate was increased by two percent until the pressure reached beyond 60 psi.

Measurements were also taken to determine percent compression of the polymers under pressure by measuring the height of the resin in the column. Due to equipment constraints, backpressure measurements were not collected. Impermeability under pressure was interpreted as destruction of the imprinted sites and collapse of the polymeric structure.

### 2.6. Unary Ion Gravity Retention Columns for Imprinted and Non-Imprinted Polymers

One hundred milligrams of imprinted polymers were swollen for 20 min using DIUF water before placement into the column. Afterwards, 10 mL of 20, 40, 60, or 80 ppm of aqueous Zn^+2^ solution was added. Each concentration of zinc was tested individually per resin derivative, in triplicate. Resins were not repeatedly exposed to varying concentrations of aqueous zinc, per trial run. The eluent was collected and subsequently diluted to a maximum concentration of 2 ppm. All eluent was analyzed via flame AA and absorbance values were compared to a standard curve to calculate remaining zinc content.

### 2.7. Binary Ion Gravity Retention Columns for Imprinted and Non-Imprinted Polymers

For gravity columns containing solutions of two metal ions all equipment and procedures were identical to those described in Section 2.6. Binary ion studies were run using aqueous solutions containing calcium chloride and zinc chloride in the following concentrations; 20:80, 40:60, 60:40, and 80:20 ppm (Zn:Ca). Each collected eluent sample was again diluted to 20 mL at a concentration of 2 ppm for samples being analyzed for Zn by flame AA or to 3 ppm for samples being analyzed for Ca. Two aliquots were taken from each eluent sample and diluted separately.

### 2.8. Infrared Spectroscopy Analysis

All infrared spectroscopy (IR) data were acquired using a Thermoscientific Nicolet (Pittsburgh, PA, USA) with iS50 ATR with solid state spectra samples being gathered using attenuated total reflection (ATR) mode. Preparatory work for samples included lyophilization and dialysis if imprinted.

### 2.9. Raman Spectroscopy Analysis

RAMAN spectra were acquired using a Kaiser Optical Systems (Ann Arbor, MI, USA) RNX1 Hololab Raman spectrometer and microscope with 4 cm^−1^ spectral bandpass and a 1024 × 256 pixel CCD detector (Andor Technology, Belfast, North Ireland). The laser source used was a Coherent DPSS (532 nm) with a 10 s exposure and 10 accumulations.

### 2.10. Thermal Gravimetric Analysis

Thermal Gravimetric Analysis (TGA) spectra were attained by TA Instruments TGA Q500 (New Castle, DE, USA) using a platinum pan with sample weights varying 5–15 mg. Instrument parameters conducted experimentation under an inert nitrogen atmosphere and before analysis was performed samples were dried within the TGA to remove excess water by ramping to 160 °C at 10 °C/min and holding said temperature for 45 min. Once dry, analysis was conducted using a ramp cycle at 10 °C/min to 700 °C.

### 2.11. Differential Scanning Calorimetry

All Differential Scanning Calorimetry data were gathered using a TA Instruments DSC Q2000 instrument (New Castle, DE, USA) with Perkins Elmer (Waltham, MA, USA) standard aluminum pans. Subsequent analysis was performed using a heat/cool cycle comprised of equilibrating the sample at (−25 °C) succeeded by ramping at 260 °C at 10 °C/min, then ramping back to −25 °C at 10 °C/min, and finally ramping back to 260 °C at 10 °C/min. The aforementioned cycle was duplicated three further times to monitor consistency in results.

### 2.12. Flame Atomic Absorption Spectroscopy Analysis

All flame atomic absorption (flame AA) data were acquired using a using a PerkinElmer Calcium-Zinc Lumina hollow cathode lamp (Waltham, MA, USA) on a flame Perkin Elmer Atomic Spectrometer 3100. The fuel to oxidant ratio used within experimentation was 2 parts zero grade air to 3 parts acetylene. Detection lamp parameters for calcium are as follows: slit width was set to 0.2 at a wavelength of 422.7 nm, with the energy output to the lamp set to 25 mA for calcium, and for zinc 213.9 nm with a slit width of 0.7.

Standardization curves for calcium and zinc were prepared from 3 ppm Ca^+2^ and 2 ppm Zn^+2^ stock solutions. Serial dilutions of the aforementioned solutions were performed to achieve 2.5, 2.0, 1.5, 1.0 and 0.50 ppm solutions.

Flame AA standard curves were developed first both for zinc and calcium ions; both achieved an *R*^2^ above 0.998. All ion solution concentrations were optimized for the specific lamp limits. For zinc, the limit lies within 0.1–2 ppm, and calcium 0.1–3 ppm, thus reducing the sensitivity of the method.

### 2.13. Porosimeter Analysis

Porosity measurements were taken on a Micromeritics ASAP 2020 (Norcross, GA, USA) surface area and pore size analyzer. All samples were subjected to a degas cycle that consisted of ramping the temperature to 170 °C and leaving the sample open to the vacuum until the pressure could be reduced to below 10 μm Hg.

Once degassed, 1–2 g of each polymer were used for B.E.T. measurements. The temperature of the sample was reduced to 77 K, and nitrogen gas was injected. Nitrogen condenses on to the surface and within pore spaces in the polymeric structure. Surface sizes and diameters of the pores were approximated using calculations that consider the pressure of the system in relation to the amount of nitrogen that was placed into the system.

For detailed approximation of pore size and surface area measurements, a modified Brunauer–Emmet–Teller (B.E.T.) equation was used. The form of the B.E.T. equation is as follows:

(1) $$\frac{p}{v\left(p_{0}-p\right)}=\frac{c-1}{cv_{m}}\frac{p}{p_{0}}+\frac{1}{cv_{m}}$$

where p is equilibrium pressure, $p_{0}$ is the saturation pressure, v is the total volume of gas adsorbed, $v_{m}$ is the monolayer gas volume, and c is the B.E.T. constant.

Measurements using this equation will be more accurate than calculations made using the Langmuir equation and thus are used in this research. It must be noted that, in B.E.T. measurements, pores of sizes of greater than 50 nm are not considered pores anymore and instead are added to the surface area value [17].

### 2.14. Scanning Electron Microscopy

Scanning electron microscopy (SEM, Hitachi VP-SEM S-3400N, Tokyo, Japan) was used to characterize morphology via the secondary electron-imaging function at 5 kV with a probe current of 6 × 10^−11^ amps and a working distance of 5–7 mm. Prior to imaging, samples were sputter coated with a 60:40 mixture of gold:palladium for three minutes to obtain contrast using a Denton Desk II (Moorestown, NJ, USA) sputter coater.

## 3. Results and Discussion

**Figure 1:**
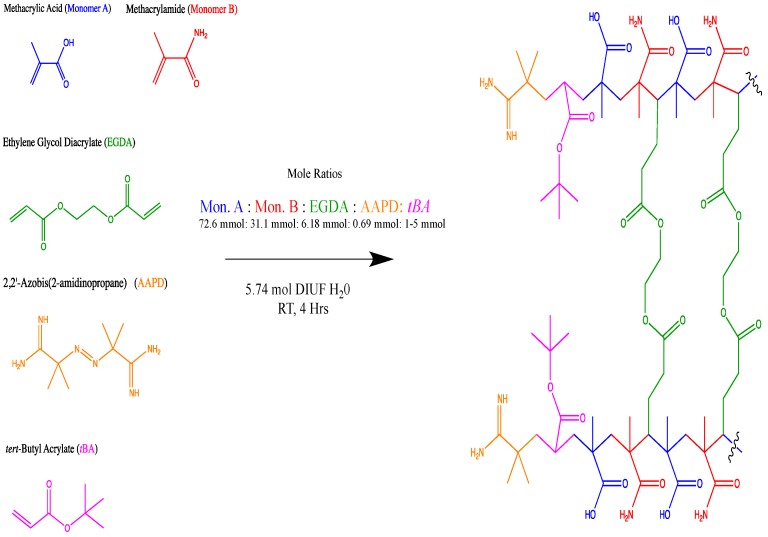
Purposed repeating unit upon ideal arrangement. Shown synthetic scheme is for non-imprinted resins.

Crosslinked resins with three different crosslinking densities (5, 10, and 15 mol %) without the co-monomer *tert*-butyl acrylate (tBA) were synthesized (Figure 1). These were compared with resins using 5% of crosslinker containing three different concentrations of tBA co-monomer (1, 3, and 5 mol %). All polymeric derivatives underwent analytical characterization, ionic retention studies, and pressure retention studies, and were subject to lyophilization before analysis. IR spectroscopy was used for quality control, as well as to monitor full conversion of monomer to polymer, water content, and intensity of hydrogen bonding.

IR and Raman spectroscopy were also used to investigate phase separation of the hydrophobic tBA co-monomer from the hydrophilic resin. Thermal gravimetric analysis (TGA) and differential scanning calorimetry (DSC) were used to monitor polymer degradation and phase transitions. This gives us an in-depth look at how crosslinking, the addition of tBA, and imprinting affect the morphology of the polymer in combination with the IR data.

Pore sizes, surface area, and pore volume are highly dependent on crosslinking density and phase separation and were measured using B.E.T. measurements. These data can be combined with TGA and DSC data to explain what is occurring at the molecular level in relation to pressure stability and zinc affinity. This information was also used in conjunction with AFM imaging to determine at a molecular level the pore size, shape, and dispersity of the polymer.

### 3.1. Infrared Spectroscopy

#### 3.1.1. IR Spectroscopy-Variable Crosslinker Study

A key characteristic noted in the IR spectra of all polymers is the presence of water remaining after lyophilization, as seen as a bigger OH stretch at 2800–3500 cm^−1^. The amount of water remaining in the polymer decreases with increasing crosslinker density (Figure 3, from top to bottom). This indicates that more crosslinker will limit both zinc’s and water’s ability to access pores within the polymer. Hydrogen bonding can be determined via IR as well. Methacrylic acid contains a carboxylic acid C=O bond whose spectral signature is a sharp, strong peak between 1710–1780 cm^−1^. Methacrylamide contains an amide C=O whose spectral signature appears as a sharp, strong peak within 1630–1690 cm^−1^ [23,24].

A noticeable trend is the difference in preference of the carboxylic acid or the amide for use in hydrogen bonding (Figure 3). When comparing the 5 mol % EGDA non-imprinted polymer to the imprinted, the intensity of the two carbonyl peaks is relatively similar. In the 10 and 15 mol % non-imprinted vs. imprinted comparisons, increasingly the carboxylic acid peak is stronger than the amide peak. This trend shows a clear preference for hydrogen bonding using the carboxylic acid over the amide when the amount of crosslinker contained within the polymer is increased, which is an indication for the more hydrophobic amide peak phase separating from the more hydrophobic sites, thus reducing access to hydrogen bonding.

#### 3.1.2. IR Spectroscopy-Variable tBA Study

In the comparison between non-imprinted and imprinted polymers containing variable concentrations of tBA (Figure 4a,b), high amount of water is still retained by the imprinted polymers after lyophilization. This is likely due to the zinc ion speciation. Zinc chloride preferentially forms zinc hexahydrate when dissolved in water [25]. Therefore, it is likely that water ligands imprinted into the polymer structure as well, and structural water is more difficult to remove.

When comparing the C=O peaks from the carboxylic acid and the amide in the non-imprinted polymers to the one in the imprinted polymers (Figure 4b), the ratio of the C=O peaks follow the same trend when increasing the concentration of tBA as found when increasing the amount of crosslinker (Figure 3). As the amount of tBA is increased, the carboxylic acid peak grows in intensity relative to the amide peak, showing that the carboxylic acid becomes more preferential for hydrogen bonding. Once the polymer is imprinted the reverse trend is seen (Figure 5, bottom). With the imprinted 1 mol % tBA polymer, the amide peak is more intense than the carboxylic acid peak. This trend continues to increase with the amount of tBA in the polymer, with the trend beginning to favor the amide greatly once the concentration of tBA reaches 5 mol %.

Thse data suggest that when the amount of tBA is increased, phase separation becomes more likely. The data also suggest that the phase separation is increased by the presence of zinc ions during imprinting. That phase separation is the main cause for the changes in the IR spectra is further strengthened by the following data.

### 3.2. RAMAN Spectroscopy

The same set of polymers used in Section 3.1 were also subjected to RAMAN analysis. The same increase in carboxylate intensity for non-imprinted materials can roughly be observed along with the increase in amide intensity for imprinted materials seen within Figure 6. The carboxylate (C=O) peaks are shown to have a RAMAN shift of roughly 1710–1755 cm^−1^, C-N amide of 740–850 and 1000–1250 cm^−1^, and OH of 3000–3600 cm^−1^. Raman, however, more strongly shows the peaks for the hydrophobic backbone of the polymer. These peaks do not show any shift with tBA concentration. This indicates that the changes in structure mainly occurs in the side chains [24,26]. The peak at 3000 cm^−1^, however, increases with tBA concentration, suggesting that the co-monomer is incorporated into the polymer structure at increasing amounts.

### 3.3. Thermogravimetric Analysis

TGA analysis was used primarily to determine purity and the consistency of the syntheses of the various polymers tested throughout the described experiments. All TGA experiments performed showed similar results. Water loss is seen around 100 °C followed by another weight loss of first the side chains and then the backbone itself (Figure 7 and Figure 8 below).

### 3.4. Differential Scanning Calorimetry

DSC analysis was used to determine the glass-transition temperature (*T*_g_) of all polymer variants. Both the duration of the transition and the difference in transition temperatures across polymers were analyzed. Looking first at the variable crosslinker study, the 5 mol % EGDA polymers differ greatly in the duration of the *T*_g_ regarding non-imprinted vs. imprinted (Table 1). The imprinting process shortens the *T*_g_ by zinc ion binding site moving several polymer chains together to form one imprinted site, reducing free volume and sharpening the glass transition. The non-imprinted polymer has more freedom of movement and so the *T*_g_ is more broad.

This trend disappears as more crosslinker is added to the structure, which is expected. Crosslinker reduces the size of polymer chain that can move.

It was found in the tBA polymers that an increase in the amount of tBA in the non-imprinted polymers caused an increase in the duration of the *T*_g_. This can be explained by the added steric hindrance of the *tert*-butyl moiety. The *tert*-butyl group prevents packing of the polymer chains. This leads to the polymer chains to have more freedom of movement.

Once the polymer is imprinted, the *T*_g_s follow the same trend. The one exception is the 5 mol % tBA polymer, whose *T*_g_ dropped lower than the 3 mol %. This indicates that the combination of imprinting and increased *tert*-butyl groups can lead to more phase separation as also indicated by the IR study.

### 3.5. Scanning Electron Microscope Imaging and Porosimeter Data

SEM imaging was carried out to further elucidate the morphology differences between imprinted and non-imprinted polymers. Only two samples were chosen for imaging, the imprinted and non-imprinted 5 mol % EGDA resin with 3 mol % tBA.

The imaging showed that imprinting changes the overall morphology of the resin. In Figure 9a, the non-imprinted resin shows some larger nodule-like structures, whereas the imprinted resin in Figure 9b indicates smaller, more ordered structures with larger pores in between the imprinted polymers possess a more ordered composition, which is likely due to higher order brought about by the imprinted sites, consistent with the phase separation seen in the IR and the shorter glass transitions seen in the DSC.

The SEM images also show that particle size decreases with imprinting. This is consistent with the B.E.T. surface area values (Table 2). Pore volume and pore size is also somewhat bigger for the imprinted resin. The ion capacity data, however, suggest the opposite. This is likely because the porosimeter measurement does not differentiate between hydrophilic and hydrophobic pores. Hydrophobic pores would not get access to hydrated ions; the characterization data suggest more phase separation for the imprinted resins, thus more hydrophobic pores.

### 3.6. Zinc Ion Retention Studies

Retention gravity columns were used to determine the amount of zinc that could be removed from 10 mL of 20, 40, and 80 ppm zinc solutions using approximately 100 mg of each polymer. The eluent was analyzed using flame AA.

Figure 10 shows retention by the non-imprinted polymers with varying crosslinker content. When the amount of crosslinker is increased, the amount of zinc removed by the polymer is decreased. This is likely due to less access of binding sites as discussed above. All non-imprinted variable crosslinker polymers removed more than 90% of the zinc in solutions up to 10 mL of 80 ppm zinc, showing a high retention rate regardless of the amount of crosslinking

When testing the imprinted, variable tBA polymers using the retention columns an interesting phenomenon was observed (Figure 11). Zinc retention was low for the 1 mol % tBA polymer with the polymer removing 98% of the zinc from a 20 ppm solution, but dropped to 89.9% with the 80 solution. However, moving on to the non-imprinted 3 mol % polymer no less than 99% of zinc was removed from any of the three solutions tested. Retention then dropped for the non-imprinted 5 mol % polymer starting at 99% removal for 20 ppm zinc dropping to 86% zinc removal from an 80 ppm solution. Particle size-distribution and surface area measurements will be used to attempt to explain these results.

Looking at the zinc-imprinted polymers, a trend was not directly visible in these experiments (Figure 11). All polymers in both the variable crosslinker and variable tBA studies removed more than 94% of zinc from 20 ppm solutions. At 80 ppm zinc, the amount of zinc removed ranged from a maximum of 67% with the imprinted 5 mol % tBA polymer to a minimum of 51% by the imprinted 1 mol % tBA polymer.

Concerning removal of zinc from water, it appears that the non-imprinted polymers tend to perform the best with the most efficient polymers, being the 5 mol % crosslinker polymer and the 3 mol % tBA polymer with greater than 99% of zinc being removed up to 80 ppm zinc. However, the non-imprinted polymers have only non-specific binding sites, so competition experiments with other cations will clarify whether the imprinted polymers are truly less effective.

### 3.7. Binary Ion Competition Retention Studies

For solutions containing solely zinc, Figure 12 shows that the non-imprinted variants out-performed their imprinted counterparts at nearly every concentration of zinc, with the trend increasing as the amount of zinc is increased. This is likely due to easier access of ion exchange sites than imprinted sites. In addition, imprinting can reduce porosity or pore access.

To test the specificity, a competition study with calcium ions was set up. Calcium ions were used because they have the strongest binding affinities to carboxylate groups and thus compete the strongest with zinc ions. The binary solution data again show that the non-imprinted polymers held a consistent rate of removal, meaning that the polymers are indeed acting as a cationic “sponge” indiscriminately removing all cations. Competition studies with calcium showed that when solutions contain predominately calcium, the zinc removal rate for imprinted polymers is reduced to 50% or below due to its larger binding affinity, but when zinc is the predominant cation in solution, this phenomenon is reversed and the polymer behaves in an analogous fashion to the unary ion trends. In all cases, non-imprinted resins perform better than imprinted resins. This might be due to the different hydrophilicity or reduced access to site seen before.

Figure 13 details the dilution values for calcium recovered from the binary ion experiments within Figure 12. If all sites were just ionic sites (i.e., imprinting would not have worked), calcium binding should stay high and consistent for all polymers (unless there is a severe difference in surface area or binding site access). This is not the case, however, indicating that there are Zinc-imprinted sites.

### 3.8. Pressure Stability Analysis

**Figure 2:**
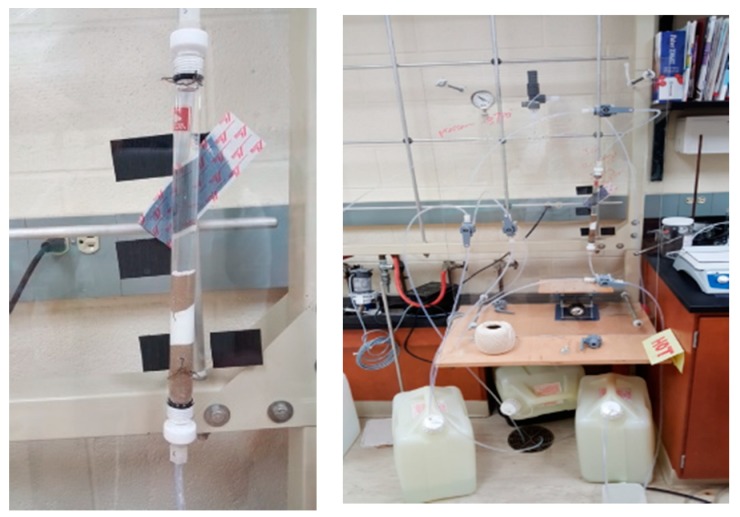
Full pressure apparatus shown on the right. Left image shows a packed column using sand and ion exchange resin.

**Figure 3:**
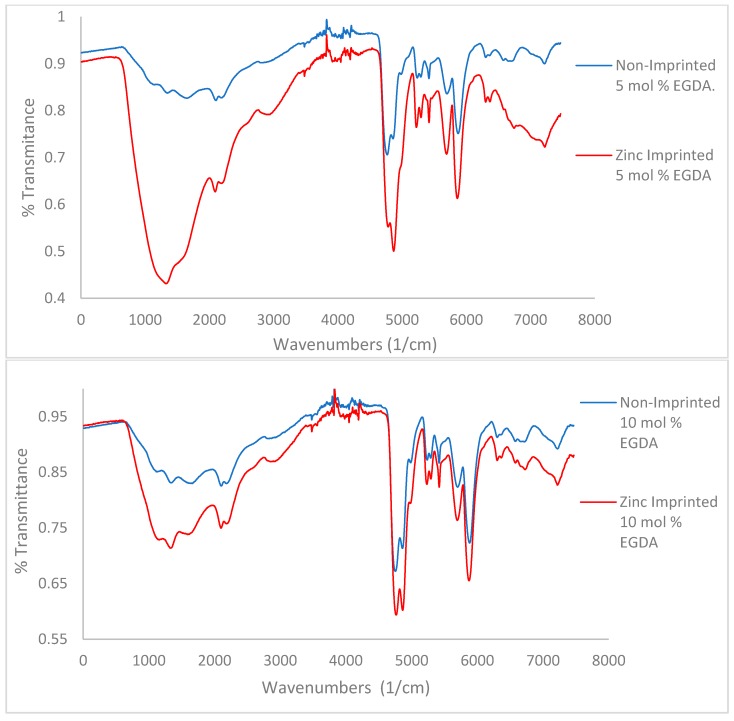

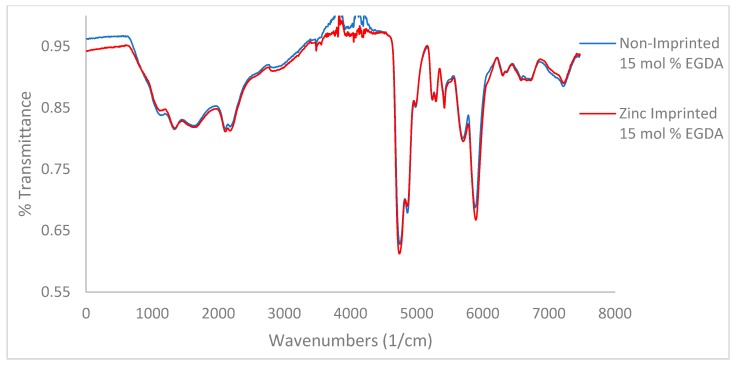
IR spectra for variable crosslinker (EGDA) study. Top to bottom 5, 10, and 15 mol %. No tBA present.

**Figure 4:**
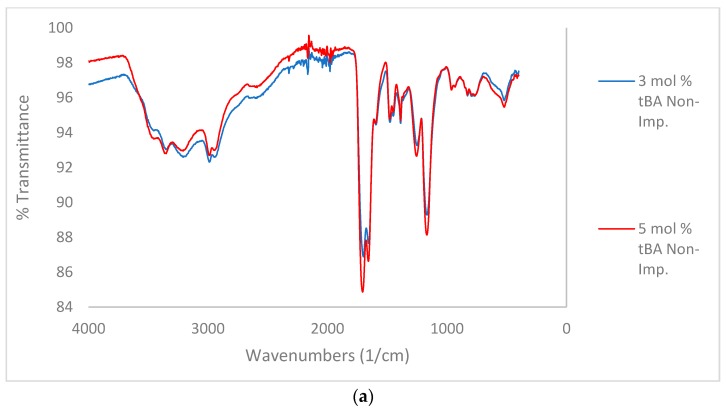

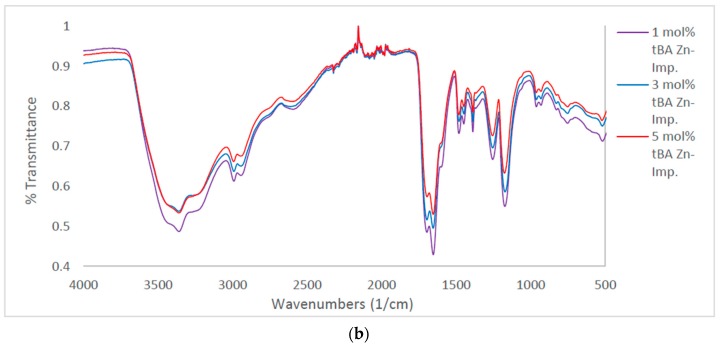
(**a**) Comparison of IR for non-imprinted resins with variable tBA. Spectra stacked in order of increasing tBA concentration. From top to bottom, spectra are 3, and 5 mol % tBA, respectively. (**b**) Comparison of IR for zinc-imprinted (bottom) resins with variable tBA. Spectra stacked in order of increasing tBA concentration. From top to bottom, spectra are 1, 3, and 5 mol % tBA, respectively.

**Figure 5:**
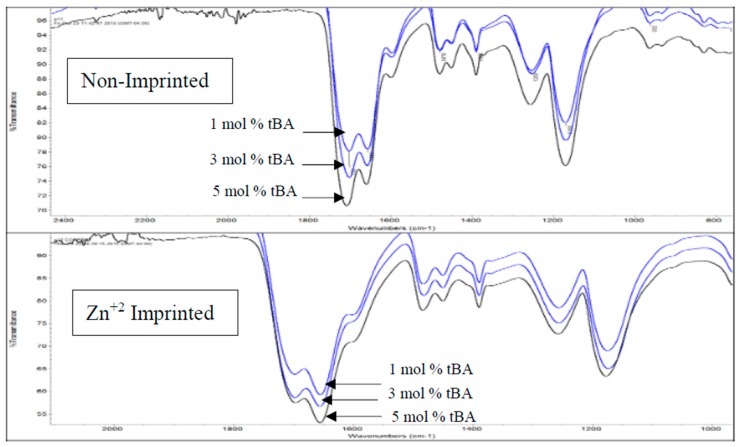
IR expansion of carboxylic acid and amide C=O region for variable tBA content. Spectra stacked in order of increasing tBA concentration with non-imprinted on top and imprinted on bottom.

**Figure 6:**
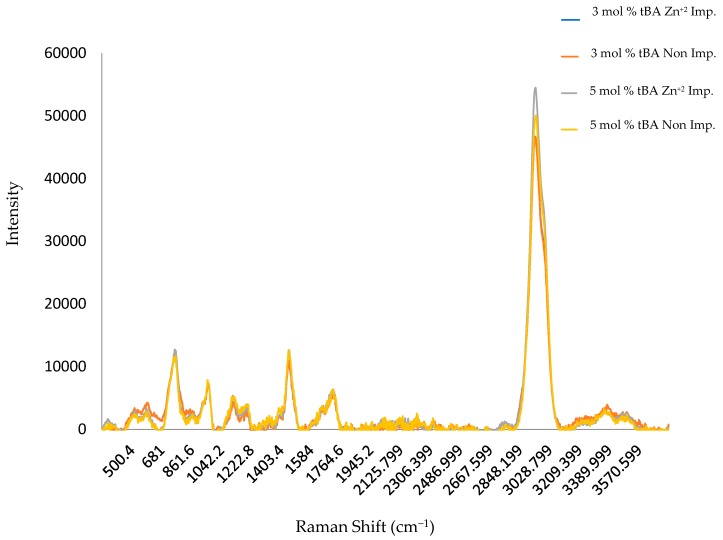
Compiled RAMAN spectra for non-imprinted and zinc imprinted polymer variants. All displayed variants contain 5 mol % EGDA crosslinker.

**Figure 7:**
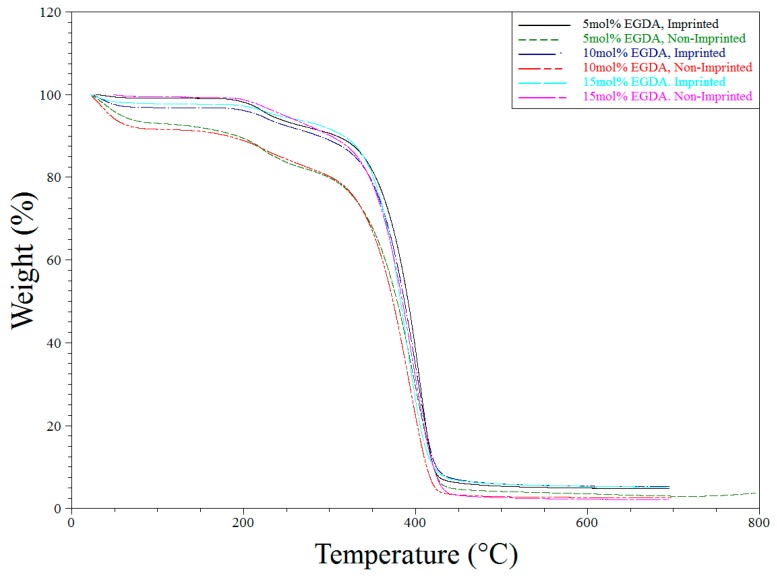
TGA degradation of all polymers synthesized for the variable EGDA study.

**Figure 8:**
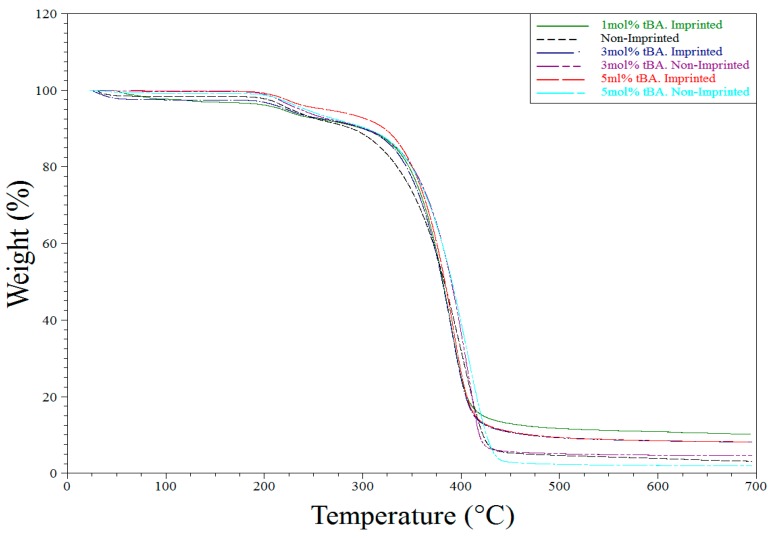
TGA degradation of all polymers synthesized for the variable tBA study.

**Figure 9:**
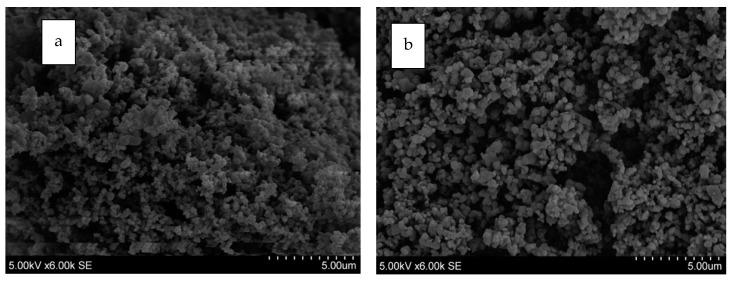
SEM mages: (**a**) non-imprinted 5 mol % EGDA, 3 mol % tBA; and (**b**) zinc imprinted 5 mol % EGDA, 3 mol % tBA. Both images are under 6000× magnification.

**Figure 10:**
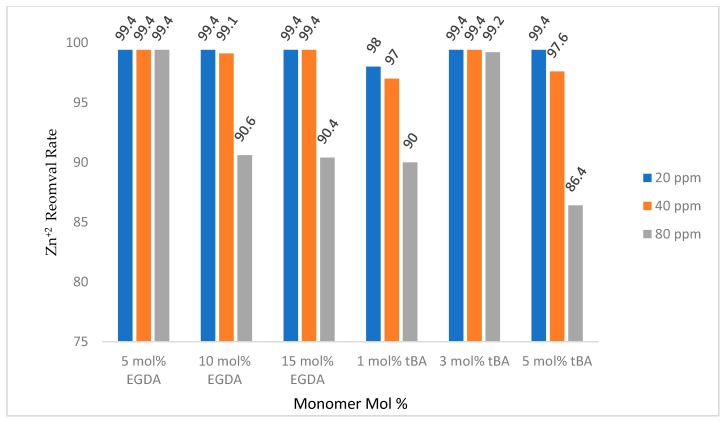
Gravity column removal rates for all tBA and EGDA non-imprinted resins. The ppm values are for Zn^+2^ solutions. All polymers with varying EGDA contain no tBA. All polymers with varying tBA contain 5 mol % EGDA.

**Figure 11:**
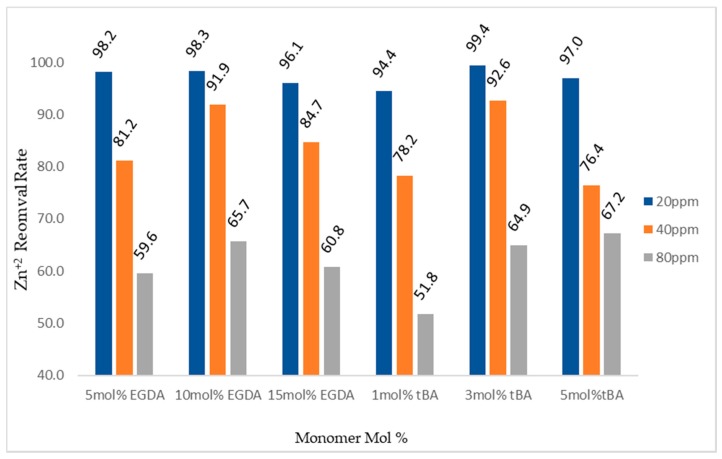
Gravity column removal rates for all tBA and EGDA imprinted polymers. The ppm values are for Zn^+2^ solutions. All polymers with varying EGDA contain no tBA. All polymers with varying tBA contain 5 mol % EGDA.

**Figure 12:**
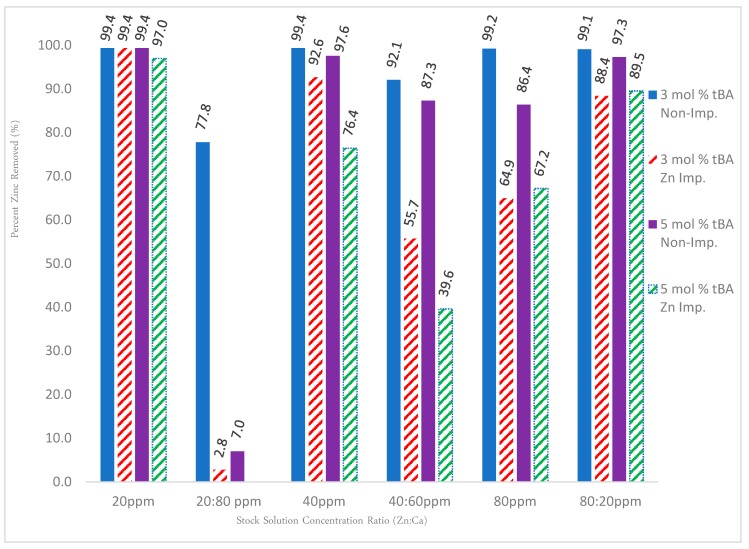
Gravity column results for both unary and binary ion studies for 3% and 5% tBA, both imprinted and non-imprinted. Removal percentages are edited to reflect the rate of removal for zinc for the binary ion studies. All polymers contain 5 mol % EGDA crosslinker.

**Figure 13:**
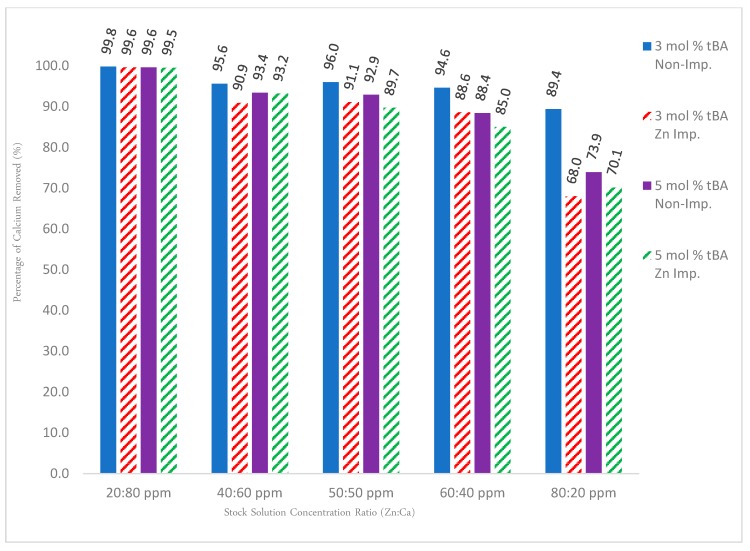
Complementary data set to Figure 12. All calcium values were obtained from the same binary ion experiments used for Figure 12. All polymers contain 5 mol % EGDA crosslinker.

The ability to place these water treatment polymers under pressure is an important aspect of this research as most water treatment facilities operate under increased pressure. Initial pressure stability measurements were used to identify the most promising polymers, which then were used for retention studies under pressure (Figure 2, left).

Approximately 1 g of each polymer was used in the pressure studies and was packed between sand after being soaked in DIUF water for 2 h. The columns were then attached to a mock water treatment set-up designed to test various materials under increased pressure.

A stroke rate of 8% was used unless otherwise noted. A polymer was considered to fail this test if the pressure in the system could not be stabilized below 60 psi or if the pressure safety valve needed to be opened. For polymers that passed, the following parameters were monitored: pressure in psi, height of the column, flow rate, and time intervals for each measurement. Once the pressure had stabilized at a given psi reading, the stroke rate was increased by 2% and pressure was monitored until stabilization occurred. Once this occurred the previously described measurements were taken once again. This process was continued until the pressure reached 60 psi, at which point the experiment was halted.

The imprinted tBA polymers performed well under pressure in comparison to their non-imprinted counterparts (Table 3). For example, the 3 mol % tBA non-imprinted polymer incurred a pressure increase from 0 psi to 25 psi over 18 min once the pump was started with a stroke rate of 10%. The pressure gradually increased beyond 60 psi over 120 min without increasing the stroke rate. When looking at the imprinted 3 mol % tBA polymer, the pressure gradually increased from 0 psi to 21 psi over two hours with a constant flow rate of 8%. This polymer then allowed the stroke rate to be increased to a maximum of 18% before reaching 55 psi. At this stroke rate the 3 mol % imprinted polymer allowed a flow rate of 52.0 mL/min, whereas the maximum flow rate achieved by the non-imprinted version was only 25.2 mL/min before reaching 60 psi with a stroke rate of 10%. That being said, the 3 mol % tBA polymers performed the best in the pressure test studies above all the others. These two polymers exhibited the slowest changes in psi at a constant stroke rate from the pump and gave the highest flow rates at high pressures relative to the other polymers tested (Table 3).

Increasing the amount of crosslinker in the structure of the polymer slightly enhanced the ability of the polymer to withstand pressures but not to the same extent as the addition of the *tert*-butyl moiety. The 5 mol % crosslinker polymers both failed to withstand increased pressure unless the safety valve was left open, even with the pump stroke rate at the instrument minimum of 5% (data not shown). Increasing the crosslinker percentage to 15 mol % helped to alleviate some of the problems, but the safety valve could not be closed without-over pressurizing the system when the non-imprinted polymer was tested (data not shown). The imprinted version of this polymer fared better; it was pressure stable for two hours with a flow rate that stabilized near 20 mL/min. However, the pressure exceeded 60 psi at the end of the experiment (data not shown).

5 mol % tBA polymers have also exhibited pressure stability (Table 4). These polymers, initially, showed similarities to the 3 mol % tBA polymers. The non-imprinted version stabilized after one hour at 55 psi with a stroke rate of 5% and a flow rate of 4.18 mL/min. Similar to the previous polymers tested, the imprinted 5 mol % tBA performed more adequately than the non-imprinted one. Nearly two hours after the pump had been started, the pressure stabilized at 15 psi with a flow rate of 20 mL/min. The stroke rate was then increased to a maximum of 14%, which gave a pressure of 44 psi and a flow rate of 37.6 mL/min. The pressure then slowly increased to 60 psi over the next 20 min, which halted the experiment. In comparison, with the 3 mol % tBA polymer the stroke rate could be increased to 18% before the pressure reached 60 psi.

Compression rates for each polymer are detailed Table 5. The amount of compression that each polymer undergoes can give insight to the stability of the pores. Affirming the results of achieved flow rates at various pressures, the 3 mol % tBA experienced the least compression under pressure. The height of the column reduced from 4.0 cm to 3.5 cm for the non-imprinted polymer, which is a 12.5% compression, and the imprinted version compressed slightly less at 11.76%. All other polymers tested experienced compressions between 24% and 28%.

## 4. Conclusions

The work described in this study expands upon earlier work in developing new ion exchange resins that are specific for a single targeted metal ion via imprinting polymerization. The target for this project was to improve pressure stability, since water treatment at water treatment facilities occurs under high pressures. At the same time, the resin is still supposed to allow for access to internal binding sites, thus increasing capacity in comparison to surface-imprinted resins with the same particle size.

These data show that the most effective way to achieve pressure stability is not to only increase the crosslinking density, but to also copolymerize with a bulky monomer at low concentrations. The data also show that the relationships between surface area and pore size, particle size, binding site access, and type of binding site (imprinted vs. ionic, hydrophilic vs. hydrophobic) are complex. The data demonstrate that, when the resin structure contains more of the hydrophobic tBA, phase separation is induced, which also creates more hydrophobic sites that are not accessible to the hydrated ions. That reduced overall ion capacity. Phase separation is also further increased with imprinting. Unfortunately, this means that selectivity can only be suggested but not proven with these initial data.

The complex relationships between resin structure and pore access will be further investigated in the future. Initial data suggest that these resins can be reused, but a detailed study will be performed in the future. This information will subsequently be used to optimize ion capacity and increase specificity, while maintaining (or improving) pressure stability and pore access.

## Figures and Tables

**Table 1:** Duration of *T*_g_ in variable tBA and crosslinker polymers.

Polymer Variant	Non-Imprinted (°C)	Imprinted (°C)
5 mol % EGDA	34 °C	24 °C
10 mol % EGDA	35 °C	37 °C
15 mol % EGDA	35 °C	32 °C
1 mol % tBA	26 °C	22 °C
3 mol % tBA	33 °C	30 °C
5 mol % tBA	44 °C	27 °C

**Table 2:** Porosity data for 3 mol % tBA Zn^+2^ non-imprinted and imprinted resins.

3 Mol % tBA Non-Imprinted			3 Mol % tBA Zn^+2^ Imprinted		
BET (m^2^/g)	Pore Volume (cm^3^/g)	Pore Size (Å)	BET (m^2^/g)	Pore Volume (cm^3^/g)	Pore Size (Å)
7.28	0.021	115	7.66	0.029	153

**Table 3:** Maximum flow rate and pressure in 3 mol % tBA imprinted and non-imprinted resins.

3 Mol % tBA Zn^+2^ Imprinted					3 Mol % tBA Non-Imprinted				
Time (min)	psi	Column Height (cm)	Flow Rate (mL/min)	Stroke Rate (%)	Time (min)	psi	Column Height (cm)	Flow Rate (mL/min)	Stroke Rate (%)
0	0	3.4	0.0	0	0	0	4.0	-	6.5
2	8	3.4	18.8	8	5	20	3.6	-	10
14	10	3.2	20.8	8	18	25	3.5	26.8	10
31	14	3.1	21.0	8	48	33	3.5	26.4	10
44	14	3.1	19.8	8	75	39	3.5	25.4	10
54	15	3	21.2	8	100	44	3.5	25.2	10
64	16	3	21.0	8	123	<60	3.5	-	
74	17	3	21.6	8					
101	18	3	21.6	8					
104	21	3	20.4	8					
107	27	3	26.4	10					
111	32	3	32.8	12					
115	39	3	38.8	14					
120	44	3	43.0	16					
120	55	3	52.0	18					

**Table 4:** Maximum flow rate and pressure in 5 mol % tBA imprinted.

5 Mol % tBA Zn^+2^ Imprinted				
Time (min)	psi	Column Height (cm)	Flow Rate (mL/min)	Stroke Rate (%)
0	0	3.3	0.0	8
3	10	2.5	28.8	8
23	10	2.5	21.2	8
86	15	2.5	20.0	8
91	18	2.5	26.6	10
96	23	2.5	33.2	12
102	50	2.5	37.6	14
108	44	2.5	0.0	10

**Table 5:** Compression and flow rate comparison of all polymer derivatives.

Polymer Variant	Percent Compression		Stabilized Flow Rate (mL/min)
1 mol % tBA	Imprinted	37.5	-
1 mol % tBA	Non-Imprinted	28.6	-
3 mol % tBA	Imprinted	11.8	43.0
3 mol % tBA	Non-Imprinted	12.5	18.0
5 mol % tBA	Imprinted	24.2	37.6
5 mol % tBA	Non-Imprinted	27.8	4.2
5 mol % EGDA	Imprinted	-	-
5 mol % EGDA	Non-Imprinted	-	-
15 mol % EGDA	Imprinted	28.9	8.4
15 mol % EGDA	Non-Imprinted	24.3	-
